# CSF1R Stimulation Promotes Increased Neuroprotection by CD11c+ Microglia in EAE

**DOI:** 10.3389/fncel.2018.00523

**Published:** 2019-01-10

**Authors:** Agnieszka Wlodarczyk, Anouk Benmamar-Badel, Oriane Cédile, Kirstine Nolling Jensen, Isabella Kramer, Nick Boe Elsborg, Trevor Owens

**Affiliations:** ^1^Department of Neurobiology Research, Institute for Molecular Medicine, University of Southern Denmark, Odense, Denmark; ^2^BRIDGE, Brain Research - Inter-Disciplinary Guided Excellence, Odense, Denmark

**Keywords:** CD11c, microglia, EAE, CCL2, IL-34, CSF1, CSF1R

## Abstract

Microglia are resident immune cells of the central nervous system. Their development and maintenance depend on stimulation of Colony Stimulating Factor-1 receptor (CSF1R). Microglia play an important role in neurodevelopment and a population of microglia that expresses the complement receptor CD11c is critical for primary myelination. This population is virtually absent in the healthy adult brain but increases dramatically upon neuroinflammatory conditions, and these microglia are suggested to play a protective role in central nervous system (CNS) diseases. To date, the molecular trigger for their expansion is unknown. Here we showed that stimulation of CSF1R by either of its ligands, CSF1 and interleukin (IL)-34, can induce expansion of CD11c+ microglia. In addition, such stimulation resulted in amelioration of EAE symptoms and decreased demyelination. Treatment with CSF1R ligands also induced expression of the chemokine CCL2, and we showed that experimental overexpression of CCL2 in the brain led to a dramatic increase of CD11c+ microglia, independent of CCR2. Moreover, this led to elevated CSF1 expression, suggesting a positive feedback loop between CSF1R and CCL2. These data provide new insights to microglia biology and open new perspectives for modulating microglial activity in neuroinflammatory diseases such as multiple sclerosis.

## Introduction

Microglia are resident macrophages of the central nervous system (CNS) that primarily mediate surveillance and immunological functions (Nimmerjahn et al., 2005; Kettenmann et al., 2011). Contrary to most tissue macrophages, microglia are long-lived yolk sac-derived cells that maintain their numbers through proliferation without contribution from bone marrow-derived precursor cells in the steady state (Ginhoux et al., 2013; Kierdorf et al., 2013; Prinz and Priller, 2014). Development, maintenance and proliferation of microglia depend on activation of Colony Stimulating Factor-1 receptor (CSF1R), a key signaling pathway in myeloid cell development (Stanley and Chitu, 2014). Activation of CSF1R by its ligands, namely Colony Stimulating Factor-1 (CSF-1) and Interleukin (IL)-34, contributes to microglial proliferation and differentiation, and is critical for microglia development, since a dramatic reduction of microglial numbers is observed in mice lacking IL-34 (Wang et al., 2012). This lack of microglia in IL-34-KO mice leads to severe developmental abnormalities in the CNS (Elmore et al., 2014), underlining the importance of microglia for neurogenesis. Other recent studies point to microglia as key players in neurodevelopment. Indeed, they participate in establishment of axonal connections (Squarzoni et al., 2014), refinement of synapses in a process called synaptic pruning (Schafer et al., 2012) and they support neuronal survival by production of Insulin-like Growth Factor-1 (IGF1) (Ueno et al., 2013).

We have recently identified a subpopulation of neonatal microglia that transiently expand soon after birth, identified by expression of the integrin complement receptor CD11c (Wlodarczyk et al., 2017). This subpopulation has a unique neurogenic and myelinogenic gene signature and is a major source of IGF1. Importantly, deletion of IGF1 in these cells leads to impaired primary myelination (Wlodarczyk et al., 2017). These findings have been supported by another study showing neonatal microglia importance in the maintenance and maturation of oligodendrocyte progenitors (Hagemeyer et al., 2017). Interestingly, CD11c+ microglia are also found to increase in numbers during neuroinflammation and neurodegeneration, such as in animal models of multiple sclerosis (MS)—experimental autoimmune encephalomyelitis (EAE) and cuprizone induced demyelination, as well as in mouse models for Neuromyelitis Optica and Alzheimer's Disease (AD) (Butovsky et al., 2006a; Remington et al., 2007; Wlodarczyk et al., 2014, 2015). We have shown that although CD11c+ microglia can efficiently present antigen to autoreactive T-cells, they are poor inducers of Th1 and Th17 responses (Wlodarczyk et al., 2014). Moreover, we showed that these microglia produce interferon (IFN)beta and high levels of IGF1 (Wlodarczyk et al., 2015), suggesting that they exert protective rather than detrimental functions in EAE. However, the exact role of CD11c+ microglia and the mechanism for their induction under neuroinflammatory conditions are not fully understood.

Here, we present new insights into the mechanism of induction of CD11c+ microglia. We show that *in vivo* stimulation of CSF1R by IL-34 or CSF1 leads to expansion of CD11c+ microglia, and that this may depend on CCL2 expression. Importantly treatment with CSF1R ligands led to suppression and reduced severity of EAE.

## Materials and Methods

### Mice

C57BL/6j bom female mice aged 7–8 weeks were obtained from Taconic Europe A/S. Female CCL2-KO and CCR2-red fluorescent protein (RFP/RFP) mice were obtained from The Jackson Laboratory and maintained in the Biomedical Laboratory, University of Southern Denmark (Odense). DBA/2-MBP/CCL2 mice that express a mouse CCL2 transgene under control of an MBP promoter (Furtado et al., 2006) were originally obtained from Bristol-Myers Squibb and maintained as a homozygous colony. 2D2 mice, which have CD4+ T cells that express a transgenic Vα3.2β11 TCR specific for myelin oligodendrocyte glycoprotein (MOG) p35-55 (Bettelli et al., 2003) were obtained from Dr. Florian Kurschus (Institute for Molecular Medicine, University Medical Center, Johannes Gutenberg-University, Mainz, Germany, now University Hospital, Heidelberg, Germany) and crossed with DBA/2-MBP/CCL2 to obtain 2D2xMBPCCL2. DBA/2-MBP/CCL2 mice were further backcrossed to C57BL/6j bom to obtain B6-MBP/CCL2. All experiments were approved by the Danish Animal Experiments Inspectorate (approval number 2014-15-0201-00369).

### EAE

Seven- to ten-week-old female mice were immunized by subcutaneous injection of 100 μL of an emulsion containing 100 μg of MOGp35–55 (TAG Copenhagen A/S, Frederiksberg, Denmark) in incomplete freunds adjuvant (DIFCO, Alberstslund, Denmark) supplemented with 400 μg heat-killed H37Ra *Mycobacterium tuberculosis* (DIFCO). *Bordetella pertussis* toxin (300 ng; Sigma-Aldrich, Brøndby, Denmark) in 200 μl of PBS was injected intraperitoneally on the same day as and the day after immunization. Animals were monitored daily from day 5 and scored on a 6-point scale as follows: 0, no symptoms; 1, partial loss of tail tonus; 2, complete loss of tail tonus; 3, difficulty walking, 4, paresis in both hind legs; 5, paralysis in both hind legs; 6, front limb weakness. Due to ethical considerations mice were sacrificed when they reached grade 6 or 24 h after hind leg paralysis.

### Intrathecal Injections

#### Adenoviral Overexpression of CCL2

Eight to 10 weeks old CCL2-deficient or CCR2-RFP/RFP mice were anesthetized by means of an intraperitoneal injection of ketamine (Ketalar®, 100 mg/kg of body weight, Intervet International B.V., Bowmeer, the Netherlands) and xylazine (Rompum®, 10 mg/kg of body weight, Bayer, Copenhagen, Denmark). Eight microliters of replication defective adenovirus (10^7^ infectious units) in sterile PBS were injected intrathecally at the site of the cisterna magna. Immediately before the injection, mice received a subcutaneous injection of buprenorphine (Temgesic®, 0.1 mg/kg of body weight, Indivior UK limited, Slough, Berkshire, Great Britain). The adenovirus encoding CCL2 was type 5 E1-E3-deleted encoding murine CCL2 gene (AdCCL2), driven by the CMV immediate-early promoter (Buschmann et al., 2012), kindly provided by Dr. J. Gauldie (Pathology & Molecular Medicine, McMaster University, Hamilton, Ontario, Canada). The adenovirus encoding the β-galactosidase gene (AdLacZ) was provided by Dr. J. Nalbantoglu (Montreal Neurological Institute, Montreal, Quebec, Canada).

Seventeen days after injection, mice were lethally anesthetized with sodium pentobarbital (Euthanimal®, 200 mg/kg of body weight, Glostrup sygehusapotek, Glostrup, Denmark), and perfused through the heart with ice-cold PBS before tissues were collected.

#### IL-34 and CSF1 Treatment

C57BL/6j mice were anesthetized by isoflurane inhalation. Fifty nanograms (Gomez-Nicola et al., 2013) of recombinant mouse IL-34 or recombinant mouse CSF1 (Biolegend, Nordic BioSite ApS, Copenhagen, Denmark) in 10 microliters of sterile PBS were injected in the cisterna magna. Analgesia was performed as described above. This treatment was performed either once or every 24 h for 3 consecutive days and the mice were sacrificed one day after the last injection. In the EAE experiments, three injections of cytokines were performed, starting at the onset of the disease (grade 2, loss of tail tonus). For each mouse, the day of first injection is referred to as day 0 (D0).

### Flow-Cytometry

Mice were anesthetized with sodium pentobarbital and intracardially perfused with ice-cold PBS. Brain tissue was collected and a single cell suspension was generated by forcing through a 70 mm cell strainer (BD Biosciences, Kongens Lyngby, Denmark). Mononuclear cells were collected after centrifugation on 37% Percoll (GE Healthcare Biosciences, Brøndby, Denmark). They were first incubated with anti-Fc receptor (Clone 2.4G2; 1 mg/ml; BD Biosciences) and Syrian hamster IgG (50 mg/ml; Jackson Immuno Research Laboratories Inc., TriChem ApS, Skanderborg, Denmark) in PBS 2% fetal bovine serum (FBS), then with anti-CD45 (Clone 30-F11; Biolegend), anti-CD11b (Clone M1/70; Biolegend), and biotin conjugated anti-CD11c (Clone HL3; BD Pharmingen) antibodies in PBS 2% FBS and finally with streptavidin-APC (Biolegend). Cell populations were gated based on isotype-matched control antibodies as CD45^dim^ CD11b^+^ CD11c^−^ (CD11c^−^ microglia), CD45^dim^ CD11b^+^ CD11c^+^ (CD11c^+^ microglia). Data were collected on an LSRII^TM^ flow cytometer (BD Biosciences) and analyzed using Flowlogic (Inivai, Victoria, Australia).

### Immunohistochemistry

#### DAB Staining

Sixteen-micrometer sections from 4% PFA-fixed, sucrose-cryoprotected frozen spinal cords of PBS-perfused mice were cut on a cryostat and stored at −20°C on Superfrost Plus slides (Fisher Scientific, Roskilde, Denmark). Sections were washed in PBS and endogenous peroxidase was depleted by incubation for 30 min in methanol + 0.2% H_2_O_2_. Endogenous biotin was blocked using Biotin Blocking system (DAKO, Agilent, Glostrup, Denmark) and incubated for 30 min in 3% BSA in PBS +0.2% Triton X-100 (Merck, Darmstadt, Germany) to prevent unspecific binding. Next, sections were incubated for 1 h at room temperature with biotinylated anti-MOG antibody (protein G affinity-purified supernatant from hybridoma clone Z2 (provided by Prof. Chris Linington, Glasgow University, UK) and IgG2a isotype control from murine myeloma) followed by 1 h incubation at room temperature with streptavidin-horseradish peroxidase (GE Healthcare Biosciences). The sections were developed using 3,3′-diaminobenzidine (DAB, 0.5 mg/ml) (Sigma-Aldrich) and H_2_O_2_ (0.033%) (Sigma-Aldrich) for 5 min and dehydrated in increasing concentrations of ethanol before clearing in xylene. Mounting was performed in Depex mounting medium (Merck).

#### Immunofluorescence Staining

Sixteen-micrometer sections from 4% PFA-fixed, sucrose-cryoprotected frozen spinal cords of PBS-perfused mice were cut on a cryostat and stored at −20°C on Superfrost Plus slides (Fisher Scientific). Sections were fixed in acetone for 10 min at room temperature followed by an incubation of 30 min at room temperature in 3% BSA in PBS +0.2% Triton X-100 to prevent non-specific binding. Next, sections were incubated for 1h with anti-NogoA (AB5664P, Millipore, Merck), and then 1 h at room temperature with anti-Rabbit IgG Alexa Fluor 488 secondary antibody (A21206, Invitrogen, Fisher Scientific), or only with anti-GFAP Cy3 conjugated antibody (C9205, Sigma-Aldrich, Merck). Slides were mounted in Fluoresence Mounting Medium (DAKO). Imaging was performed on a FV1000MPE microscope (Olympus).

#### Semi Quantitative Analysis of Demyelination

Analysis of demyelination was performed by an investigator who was blinded to the study. Five to ten parallel sections of the lumbar part of the spinal cord were graded based on the degree of loss of MOG immunoreactivity on 0–3 point scale where 0 = no lesions, 0.5 = one small lesion, 1 = 1 extended lesion; 1.5 = 2 small lesions; 2 = 2 extended lesions; 2.5 = 3 small lesions; 3 = at least 3 extended lesions. The mean values from all the sections from each animal were plotted as bar graphs.

### RT-qPCR

RNA was extracted from brain tissue using TRIzol (Invitrogen, Fisher Scientific) according to the manufacturer's protocol. cDNA was transcribed from 1 μg of total RNA using M-MLV reverse transcriptase (Invitrogen, Fisher Scientific) following the manufacturer's instructions. Quantitative real-time polymerase chain reaction (qPCR) was performed using 1 μL of cDNA combined with 24 μL of Maxima probe/ROX qPCR master mix (Fermentas, Fisher Scientific) with primers and probes (TAG Copenhagen A/S) as follows: *Itgax* (forward primer TGCAGAAGGCCAAGTATTCCTT; reverse primer CAGAGGCCCTGACTCCTGTCT; probe AAGAAAGAGGACCAGCAGT), *Ccl2* (forward primer TGGAGCATCCACGTGTTG; reverse primer ACTCATTGGGATCATCTTGCT; probe CTCAGCCAGATGCAGTT), *Ifng* (forward primer CATTGAAAGCCTAGAAAGTCTGAATAAC; reverse primer TGGCTCTGCAGGATTTTCATG; probe TCACCATCCTTTTGCCAGTTCCTCCAG), *Il17a* (forward primer CTCCAGAAGGCCCTCAGACTAC; reverse primer TGTGGTGGTCCAGCTTTCC, probe ACTCTCCACCGCAATGA), *Ifnb1* (forward primer GCGTTCCTGCTGTGCTTCTC, reverse primer TTGAAGTCCGCCCTGTAGGT, probe CGGAAATGTCAGGAGCT), *Il10* (forward primer GGTTGCCAAGCCTTATCGGA, reverse primer ACCTGCTCCACTGCCTTGCT, probe TGAGGCGCTGTCATCGATTTCTCCC). TaqMan® PreAmp Master Mix Kits were used for *Ccr4* (Mm01963217_u1, Applied Biosystems, Fisher Scientific), *Il-34* (Mm01243248_m1, Applied Biosystems, Fisher Scientific) and *Csf1* (Mm00432686_m1, Applied Biosystems, Fisher Scientific).

For *Igf1*, Maxima SYBR Green/ROX qPCR Master Mix (2X) Probe/ROX qPCR Master Mix (Fermentas, Fisher Scientific) with forward and reverse primers (800 nM; from TAG Copenhagen A/S) were used with *Igf1* primers (Forward primer CCG AGG GGC TTT TAC TTC AAC AA; Reverse primer CGG AAG CAA CAC TCA TCC ACA A). qPCRs were performed on a QuantStudio 3 apparatus (Applied Biosystems, Fisher Scientific). Results were expressed relative to 18S rRNA as endogenous control (TaqMan™ Ribosomal RNA control Reagents kit; Applied Biosystems, Fisher Scientific). One microliters of 1/1,000 diluted cDNA were used for 18S rRNA analysis.

## Results

### IL-34 and CSF1 Induce CD11c+ Microglia

Since CSF1R signaling is a potent inducer of microglial proliferation, differentiation and maintenance, we asked whether it could play a role in the generation of CD11c+ microglia.

**Figure 1:**
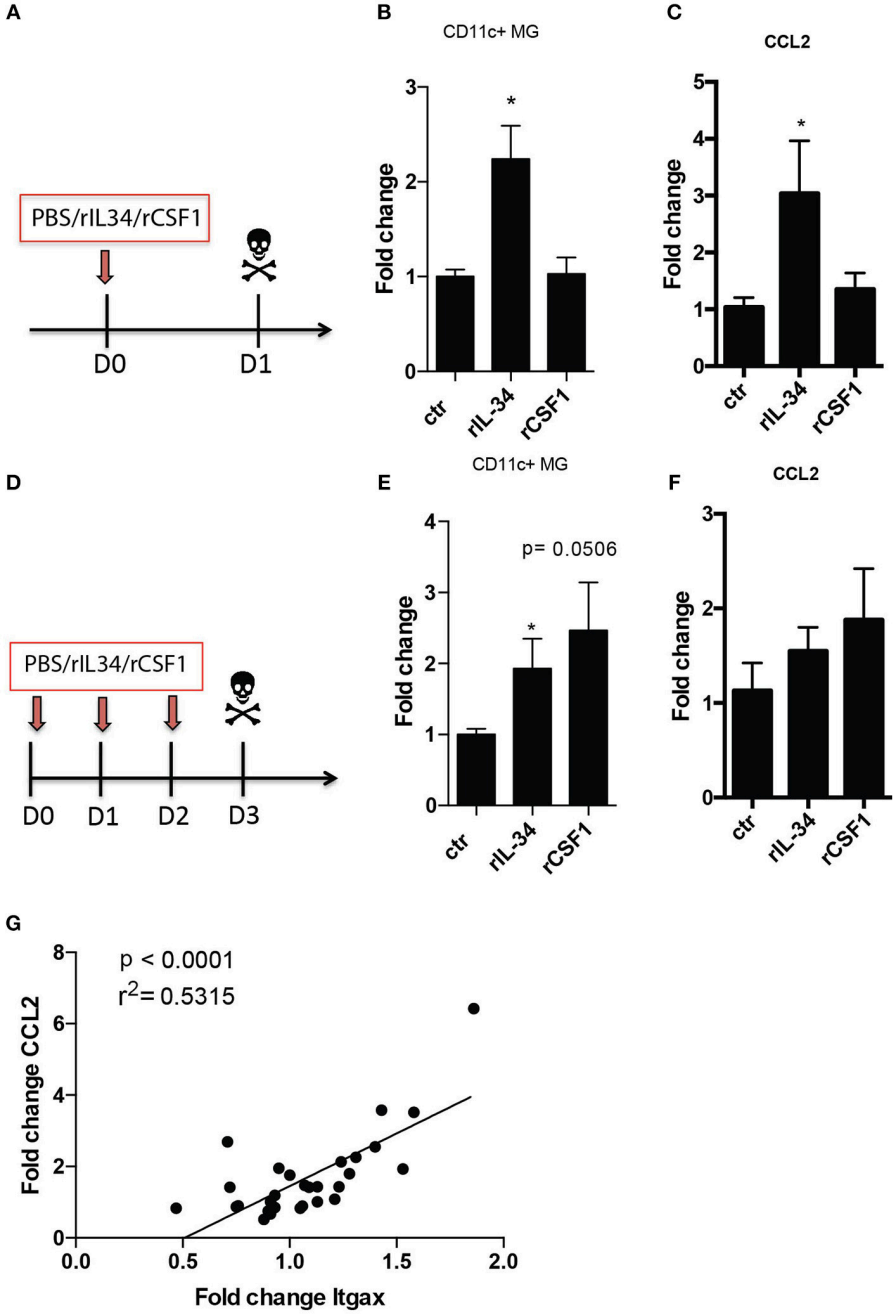
CD11c+ microglia increase due to CSF1R stimulation. B6 mice received one **(A–C)** or three consecutive **(D,E)** i.t injections of 50ng rIL34, 50ng rCSF1 or vehicle (ctr). **(B,E)** Flow cytometry analysis of brains showing fold increase of CD11c+ microglia relative to mean number in ctr animals after single (*n* = 8, *n* = 7, *n* = 9) **(B)** and three consecutive i.t injections (*n* = 6; *n* = 6; *n* = 5). **(C,F)** RT-qPCR analysis of brains showing fold increase of *Ccl2* expression relative to mean expression in ctr animals after single (*n* = 4, *n* = 5, *n* = 4) **(C)** and three consecutive i.t injections: *n* = 5; *n* = 6; *n* = 5 **(F). (G)** *Ccl2* mRNA levels positively correlated with *Itgax* gene expression in brains of mice that received one or three consecutive i.t injections of 50 ng rIL34, 50 ng rCSF1 or ctr (*n* = 28). Data are pooled from at least two individual experiments and presented as means + SEM; *p < 0.05 assessed using Mann–Whitney *U*-test.

A single intrathecal (i.t) injection of 50 ng of recombinant IL-34 (rIL-34) but not rCSF1via the cisterna magna to otherwise unmanipulated mice (Figure 1A) led to a significant increase in numbers of CD11c+ microglia (Figure 1B). We were then interested to see if repeated injections of this cytokine could further expand the CD11c+ microglia population. Three daily injections of rIL34 (Figure 1D) however sustained CD11c+ microglia increase did not cause further expansion of these cells. Three injections of rCSF1 also resulted in expansion of these cells (Figure 1E), indicating that although the pharmacokinetics of these CSF1R ligands differed slightly, their outcomes were equivalent.

The increase of the CD11c+ microglial population that has been described in neuroinflammatory and neurodegenerative models all coincidence with upregulation of the chemokine CCL2. We asked if intrathecal stimulation of CSF1R had an impact on *Ccl2* expression. Analogous to effects on CD11c+ microglial numbers, a single intrathecal injection of rIL-34 but not CSF1 resulted in significantly elevated expression of *Ccl2* mRNA in the brains (Figure 1C). We observed an increase in CCL2 expression after 3 consecutive injections of both IL-34 and CSF1 that seemed proportional to observed CD11c+ microglial numbers, although this did not reach statistical significance (Figure 1F). We then wished to clarify whether there was a correlation between CCL2 upregulation and CD11c+ microglia increase. Since neither treatment resulted in immune cell infiltration (not shown), we used CD11c gene (*Itgax*) expression as a surrogate for CD11c+ microglia expansion. We showed significant positive correlation between expression of *Ccl2* and *Itgax* mRNA in the brain (Figure 1G), indicating that CCL2 expression, however induced, could account for the increase of CD11c+ microglia.

### Overexpression of CCL2 Leads to an Increase of CD11c+ Microglia

**Figure 2:**
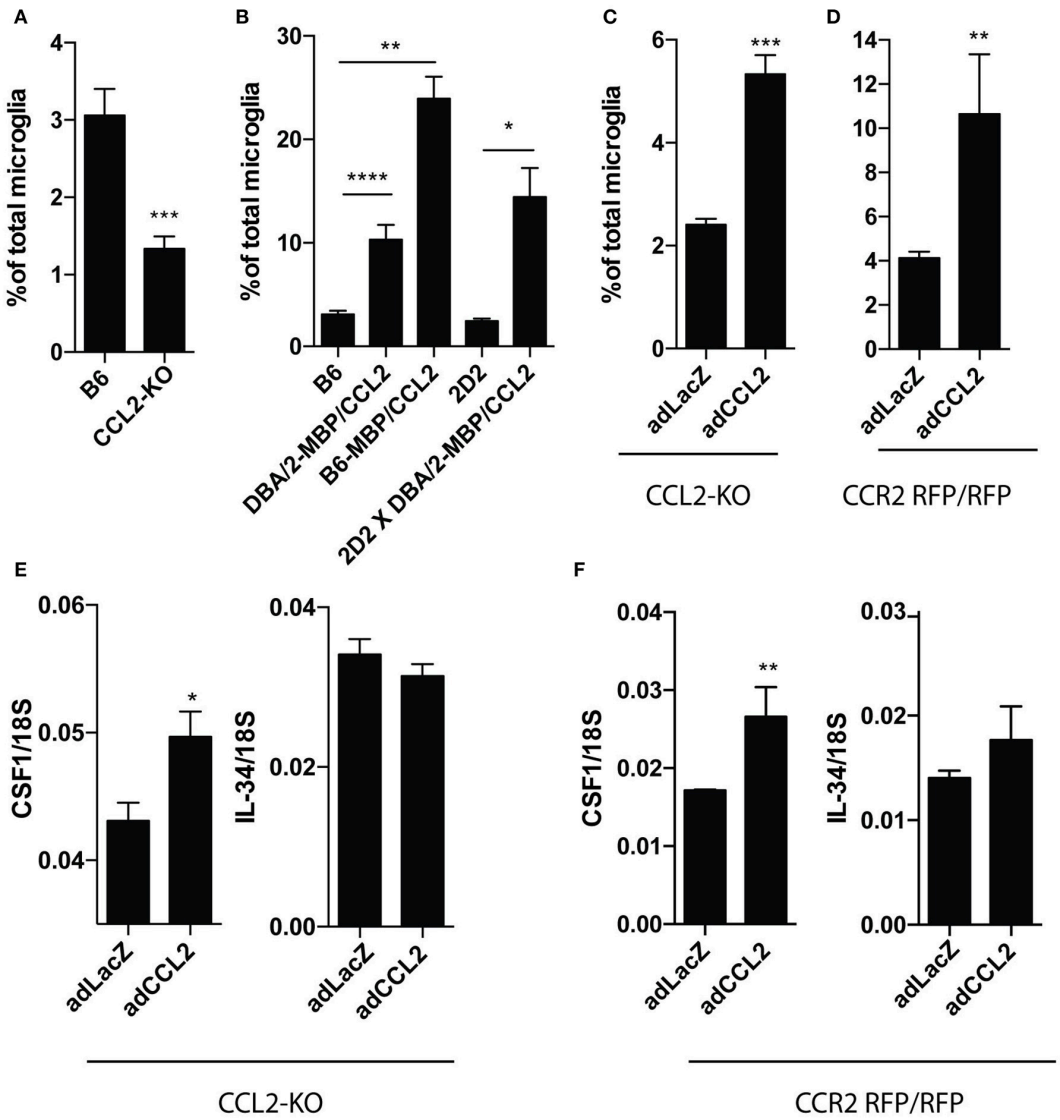
Experimental overexpression of CCL2 leads to expansion of CD11c+ microglia. Results from flow cytometry analysis showing difference in percentage of CD11c+ microglia from total microglia in brains of: B6 (*n* = 14) and CCL2 KO (*n* = 6) mice **(A)**, transgenic mice with over expression of CCL2 and corresponding control animals; B6 (*n* = 14); DBA/2-MBP/CCL2 (*n* = 8); B6-MBP/CCL2 (*n* = 3); 2D2 (*n* = 5); 2D2x DBA/2-MBP/CCL2 (*n* = 4) **(B)**, as well as in CCL2 KO (*n* = 7; *n* = 12) **(C)** and CCR2 RFP/RFP (*n* = 5) **(D)** mice infected with adenoviral vector expressing CCL2 or control vector expressing LacZ. RT-qPCR analysis of brains of CCL2 KO (*n* = 5) **(E)** and CCR2 RFP/RFP (*n* = 5; *n* = 6) **(F)** mice infected with adenoviral vector expressing CCL2 or LacZ, showing expression of CSF1 and IL34, relative to 18S. Data are pooled from at least two individual experiments and presented as means +SEM; *p < 0.05; **p < 0.01;***p < 0.001;****p < 0.0001 assessed using Mann–Whitney *U*-test.

We then asked if CCL2 plays any role in induction of CD11c+ microglia. We first compared numbers of CD11c+ microglia in the brains of CCL2-deficient and wild-type (WT) mice. Proportions of CD11c+ microglia were significantly reduced in CCL2-deficient brains (Figure 2A). We subsequently investigated CD11c microglia proportions in the brains of transgenic mice that overexpressed CCL2 under control of an MBP promoter, on three strain backgrounds (DBA/2-MBP CCL2, DBA/2-MBP CCL2 × 2D2, and B6-MBP-CCL2). Brains of all of these mice contained elevated proportions of CD11c+ microglia, reaching almost 25% of total microglia in case of B6-MBP-CCL2, nearly a 10-fold increase as compared to levels in non-transgenic B6 mice (3%) (Figure 2B). We then asked if compensating for lack of CCL2 in CCL2-KO mice by induction of CCL2 expression with an adenoviral vector would restore proportions of CD11c+ microglia. Figure 2C shows that this intervention resulted in significantly higher proportion of CD11c+ microglia compared to control virus-infected CCL2-KO mice. To assess whether the induction of CD11c+ microglia was dependent on the CCR2- receptor for CCL2, we overexpressed CCL2 in CCR2 RFP/RFP mice that lack functional CCR2. This overexpression also led to a significant increase of CD11c+ microglia (Figure 2D). This increase was noticeably higher than in transfected CCL2-KO, which could be explained by higher overexpression of adenovirus-encoded CCL2 in CCR2 rfp/rfp mice as we previously reported in Cedile et al. (2017). Interestingly, expression of CCL2 in CCL2-KO (Figure 2E) as well as CCR2 rfp/rfp (Figure 2F) brains resulted in a significant upregulation of *Csf1* but not *Il34* mRNA levels, suggesting a positive feedback loop between CCL2 and CSF1.

### Stimulation of CSF1R Leads to Amelioration of EAE

**Figure 3:**
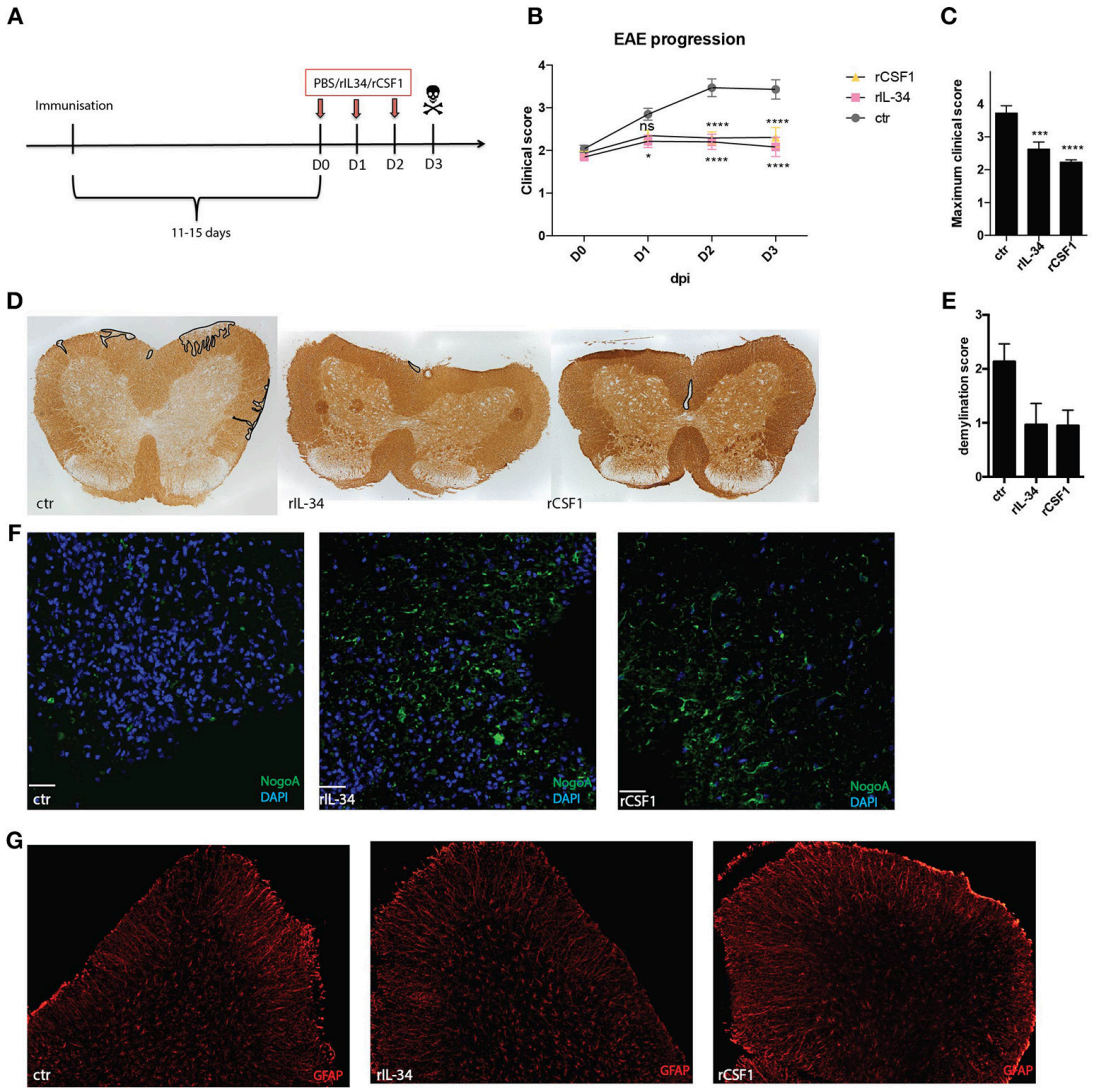
CSF1R stimulation ameliorates EAE. Mice were immunized with MOG35-55+CFA and pertussis toxin. At the onset of the disease (D0, typically 11–15 days post immunization) mice received three consecutive i.t injections of: 50ng rCSF1 (*n* = 16); 50 ng rIL-34 (*n* = 20) or ctr (*n* = 22) at D0, D1, D2, and were sacrificed at D3 **(A)**. Graphs show EAE progression **(B)** and maximum clinical score **(C)** for these mice. Data are pooled from eight individual experiments *p < 0.05; ***p < 0.001;****p < 0.0001 assessed using two-way ANOVA **(A)** or student's *t-*test **(B). (D,E)** Representative micrographs **(D)** and semi-quantitative assessment **(E)** of demyelination (defined as loss of MOG immunoreactivity in spinal cord). **(F,G)** Representative micrographs showing NogoA+ oligodendrocytes (green) **(F)** and GFAP+ astrocytes (red) **(G)** in spinal cord. Data are pooled from at least three individual experiments (*n* = 3; *n* = 3; *n* = 4).

To investigate the role of CD11c+ microglia in neuroinflammation we treated B6 mice that showed first signs of EAE (typically 11–15 days post immunization) daily for 3 consecutive days with 50 ng of rIL-34 or 50 ng of rCSF1, delivered to the cisterna magna as before (Figure 3A). Both cytokines effectively suppressed disease progression (Figure 3B) and reduced disease severity (Figure 3C). The effect was already significant 24 h after first injection of rIL34 and 24 h after second injection of rCSF1. Histological analysis revealed reduced demyelination (Figures 3D,E) and reduced loss of oligodendrocytes (Figure 3F) in both treated groups in comparison to vehicle-treated controls. We observed no difference in astrocyte activation measured as GFAP gene expression (not shown) and no morphological changes (Figure 3G).

**Figure 4:**
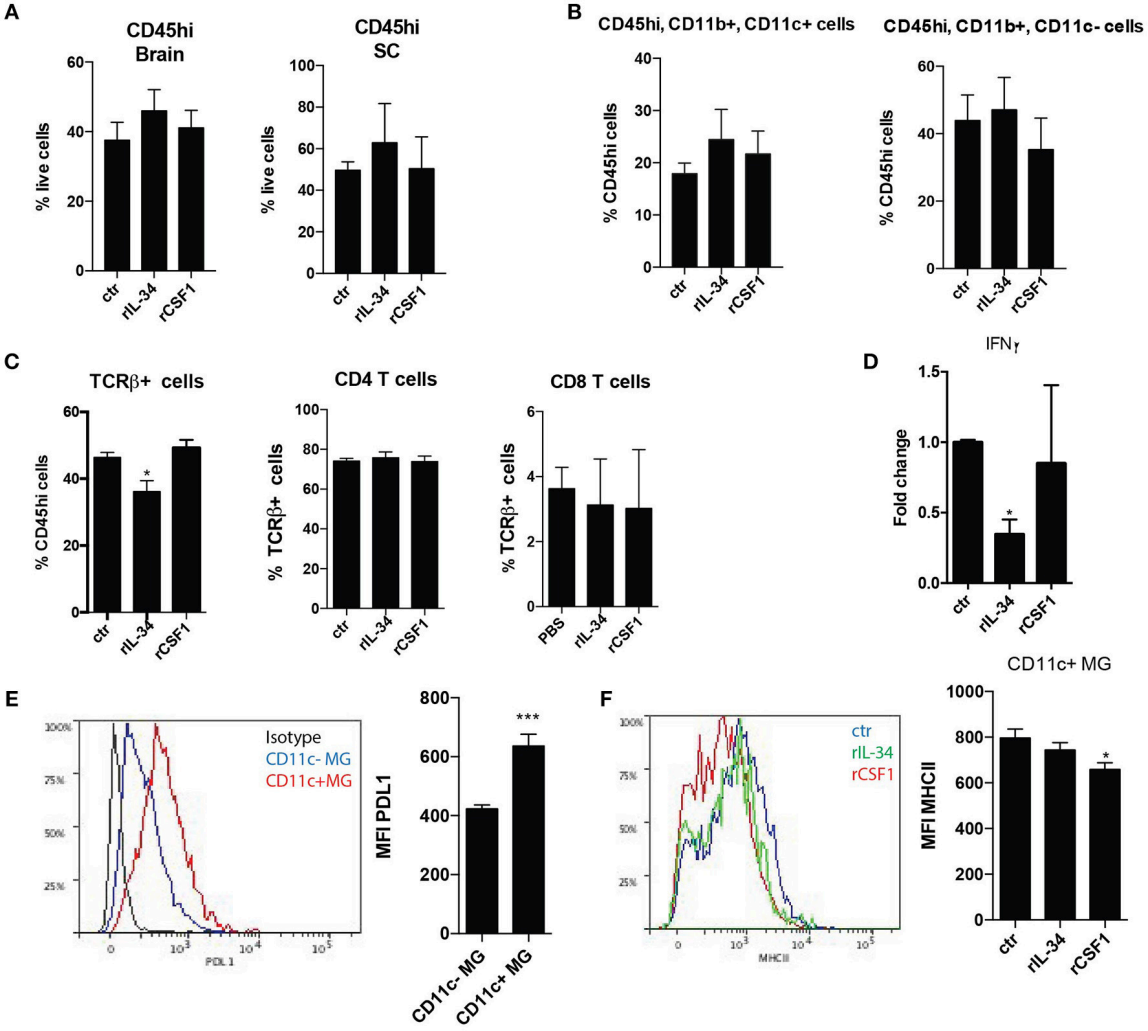
CSF1R stimulation in EAE has no impact on leukocyte infiltration but regulates microglia response. **(A–C)** Flow cytometry analysis of CNS of mice with symptomatic EAE that received three consecutive i.t injections of: 50 ng rCSF1 (*n* = 3), 50 ng rIL-34 (*n* = 4), or ctr (*n* = 6) at D0, D1, and D2 (as in Figure 3), showing percentage of infiltrating immune cells gated as CD45high from a live gate in brain and SC **(A)**, and proportions of CD11b+ CD11c+/- myeloid cells **(B)** as well as TCRbeta+ T-cells from CD45high cells and proportions of CD4+ and CD8+ T-cells from CD45high TCRbeta+ cells in the SC **(C). (D,E)** RT-qPCR analysis showing *Ifng* expression in brains: (*n* = 4, *n* = 6, *n* = 5) **(D)**. Representative histogram showing PDL1 expression on CD11c+ (red) and CD11c– (blue) microglia defined as CD45low CD11b+ and an isotype control (black) as well as bar graph showing MFI of PDL1 expression **(E). (F)** Representative histogram showing expression of MHCII in CD11c+ microglia from mice with symptomatic EAE that received three consecutive i.t. injections of: 50 ng rCSF1 (red), 50 ng rIL-34 (green) or ctr (blue) as well as bar graph showing MFI of MHCII **(F)**. Data are pooled from at least 3 individual experiments, presented as means +SEM *p < 0.05; ***p < 0.001, assessed using Mann–Whitney *U*-test.

Flow-cytometric analysis of the CNS of these mice showed that neither the percentage of infiltrating immune cells (Figure 4A) nor the composition of myeloid cells were affected (Figure 4B, not shown). However, there was a significant decrease in TCRbeta+ T cells in the spinal cord (SC) upon rIL-34 treatment (Figure 4C). Neither treatment induced significant effect on gene expression of pro- (GM-CSF, IL17) or anti-inflammatory (IFNbeta, IL10, IGF1) cytokines in the CNS (not shown), although there was a significant reduction of *Ifng* mRNA levels in the brains of mice that were treated with rIL-34 (Figure 4D). Interestingly, CD11c+ microglia expressed higher levels of PDL1 than their CD11c- counterparts (Figure 4E), and this was not affected by treatment (not shown), whereas treatment with rCSF1 but not rIL-34 led to a small but significant decrease of MHCII expression on CD11c+ microglia (Figure 4F).

## Discussion

In this study we show that CSF1R stimulation in the CNS by its ligands leads to expansion of the CD11c+ microglia subpopulation. Moreover, this population of microglia can also be induced by overexpression of the chemokine CCL2. Interestingly, CSF1R stimulation upregulated CCL2 expression, which correlated with CD11c+ microglia expansion. Moreover, we demonstrated that treatment with CSF1R ligands IL-34 and CSF1 dramatically ameliorated EAE symptoms and reduced demyelination.

The role of microglia in neuroinflammatory conditions such as in EAE is still unclear. Their activation is a hallmark of demyelinated lesions in the MS brain and microglial activity correlates with progression of disability (Rissanen et al., 2018). In EAE, microglial cells were reported to participate in disease aggravation by re-activating T cells that had entered the CNS (Aloisi et al., 1998). Depletion of microglia in EAE proved efficient to repress symptoms (Heppner et al., 2005; Nissen et al., 2018). These findings implicated microglia with negative outcomes in the disease. However, beneficial effects of microglial cells in models of MS have also been reported. In response to demyelination microglia produced anti-inflammatory IFNbeta and cleared myelin debris, so facilitating the process of remyelination that leads to amelioration of the disease (Yamasaki et al., 2014; Kocur et al., 2015). It has also been shown that transplantation of microglia pre-stimulated with IL-4 into the CSF resulted in oligodendrogenesis and led to amelioration of EAE (Butovsky et al., 2006b). Recently we have demonstrated that CD11c+ microglia do not polarize T-cells toward a pathogenic phenotype but in turn express neuroprotective and anti-inflammatory factors such as IGF1, SPP1 and IFNbeta (Wlodarczyk et al., 2014, 2015, 2017). Our data here further support an anti-inflammatory phenotype of CD11c+ microglia, showing that they express higher levels of PDL1 in comparison to their CD11c- counterparts. We have previously shown that during EAE both CD11c+ and CD11c- microglia express equal levels of CSF1R and that they expand due to proliferation (Wlodarczyk et al., 2015). It has been shown that IL-34, unlike CSF1, induced neuroprotective properties of microglia in an AD model (Du Yan et al., 1997; Mizuno et al., 2011). This suggests that microglial phenotypes may differ depending on CSF1R ligand stimulation. Here we showed that treatment with CSF1 can modulate phenotype of CD11c+ microglia by decreasing their expression of MHCII. Stimulation of CSF1R by both ligands had a beneficial effect on EAE progression. The faster kinetics in case of rIL-34 treatment is consistent with a delayed induction of CD11c+ microglia by rCSF1. Amelioration of EAE symptoms after rIL-34 and rCSF1 treatment with concomitant reduction of demyelination is in line with a recent study showing beneficial effects of CSF1 in cuprizone-induced demyelination. This intervention led to microglial activation, upregulation of IGF1 as well as reduction of demyelination (Laflamme et al., 2018). Although Laflamme and colleagues showed only marginal effect of CSF1 treatment on infiltrating leukocytes in the cuprizone model and in our model there were no obvious differences in leukocyte composition, CSF1R is broadly expressed on monocytes/macrophages and we cannot exclude that the CSF1 and IL-34 treatment modulated response of such cells.

CD11c+ microglia are virtually absent in the healthy adult brain, and are mobilized in response to inflammatory cues, demyelination, neurodegeneration (Butovsky et al., 2006a; Remington et al., 2007; Wlodarczyk et al., 2014, 2015). Interestingly, levels of the chemokine CCL2 were elevated in the disease models where these cells have been described such as EAE, cuprizone induced demyelination as well as AD (Mahad and Ransohoff, 2003; Janelsins et al., 2005; Buschmann et al., 2012). Our data show that CCL2 expression positively correlated with expression of *Itgax* mRNA and thus CD11c+ microglia expansion and we could demonstrate that overexpression of CCL2 in the brain induced significant increase of CD11c+ microglia population. Importantly we demonstrated that adenoviral vector-induced expression of CCL2 in CCL2-KO brain restored proportions of CD11c+ microglia to levels similar to those seen in WT animals. It has been reported that CSF1R ligands can induce expression of CCL2 in leukocytes (Eda et al., 2010; Zwicker et al., 2016). Here we confirm these observations showing elevated expression of *Ccl2* upon IL-34 and CSF1 treatment in the brain. This coincides with an expansion of CD11c+ microglia raising the possibility that CCL2 induction by CSF1R stimulation is directly or indirectly responsible for mobilization of CD11c+ microglia. We also show increase of *Csf1* mRNA following experimental CCL2 upregulation. This is in line with RNA-seq data showing upregulation of CSF1 expression in CD11c+ microglia (Wlodarczyk et al., 2017) and suggests a positive feedback loop between CSF1R ligands and CCL2 that mediates endogenous regulation of microglia activity. The fact that induction of these microglia was not dependent on CCR2 suggests that another receptor for CCL2 may be involved. We have previously identified CCR4 as an alternative receptor for CCL2 in the CNS (Cedile et al., 2017). Whether CCL2 acts directly on microglia via CCR4, or another cell population such as meningeal or perivascular macrophages (Prinz et al., 2017) astrocytes or neurons that also express CCR4 (Cedile et al., 2017) remains to be determined. The CCL2-CCR2 axis is generally considered to be proinflammatory, but here we propose that CCL2 induced via CSFR1 stimulation can promote CNS protection in a CCR2 independent manner.

Taken together we describe here that treatment with CSF1R ligands induces an increase of protective CD11c+ microglia and leads to amelioration of EAE symptoms and reduced demyelination. Moreover, these protective microglia are induced by CCL2 signaling. These data provide new insights in microglia biology that can open new avenues for treatment directed to modulate microglia activity in neuroinflammatory diseases such as MS.

## Author Contributions

AW, OC, and TO designed the work. AW, AB-B, IK, OC, NE, and KJ performed the experiments. AW and AB-B analyzed the data. AW, AB-B, and TO wrote the manuscript. All authors read and approved the final manuscript.

### Conflict of Interest Statement

The authors declare that the research was conducted in the absence of any commercial or financial relationships that could be construed as a potential conflict of interest.
